# How Is Daily Social Interaction Related to Loneliness in Older Adults? The Roles of Trait Loneliness and Personality.

**DOI:** 10.1093/geroni/igab046.1778

**Published:** 2021-12-17

**Authors:** Ruixue Zhaoyang, Stacey Scott, Karra Harrington, Martin Sliwinski

**Affiliations:** 1 The Pennsylvania State University, University Park, Pennsylvania, United States; 2 Stony Brook University, Stony Brook, New York, United States

## Abstract

Loneliness is prevalent among older adults and is associated with increased risks for morbidity and mortality. This study examined what types of social interactions could reduce loneliness for older adults and who would benefit the most from social interactions. We used data from 312 community-dwelling older adults (aged 70 to 90 years) who completed ecological momentary assessments (EMA) five times a day for 16 consecutive days using smartphones (n=20,507 reports), as part of the ongoing Einstein Aging Study (EAS). At each EMA, participants reported their social interactions in the past 3 to 4 hours and their current feelings of loneliness. Results from multilevel models revealed that older adults reported lower levels of loneliness on occasions when they had pleasant social interactions (p<.000) or interactions with family (p=.001) in the past few hours, compared with occasions when they had no social interaction. In contrast, they reported higher levels of loneliness if they had unpleasant social interactions in the past few hours (p=.004). These within-person (WP) effects of social interactions on momentary loneliness were significantly moderated by participants’ trait levels of loneliness and neuroticism; and were significantly stronger among those with higher (vs. lower) trait loneliness (ps <.001) or neuroticism (ps <.042). Other personality traits (Extraversion, Openness, Conscientiousness, Agreeableness) did not moderate any WP association. These results highlight the importance of having pleasant social interactions and frequent interactions with family for reducing older adults’ loneliness in daily life, especially for those higher in trait loneliness and neuroticism.

